# The True Site and Probable Causes of Placenta Prævia

**Published:** 1876-11

**Authors:** John Bartlett

**Affiliations:** Chicago


### THE TRUE SITE AND THE PROBABLE CAUSES OF PLACENTA PRÆVIA.

BY JOHN BARTLETT, M.D., OF CHICAGO.

*(Read before the Chicago Society of Physicians and Surgeons, Dec.* 27, 1875.)

By many, if not all writers, the site of the placenta, in placenta prævia, is, as I believe, erroneously given, and the cause of its abnormal position is shrouded in too deep an obscurity. The present paper is an attempt to show that there is nothing very singular in the location of the presenting placenta, and that this malposition is one of the simplest *errores loci* of the ovum.

In considering the subject, I shall first point out what, with due deference to the great obstetrical teachers, with whose works I am familiar, I view as a gross misconception in regard to the anatomical site of the placenta in placenta prævia.

The first obstetrician who recognized this condition was Portal; writing in 1672, he says: "I found the after-birth just before and quite across the whole *inner* orifice." In 1730 Giffard writes: "I found the placenta closely adhering around the *os internum.*" Says Smellie, in 1752: "The edge or middle of the placenta sometimes adheres to the inside of the *os internum.*" This view of the location of the placenta prævia, namely, over and above the os internum, has received, so far as I am informed, the unanimous indorsement of writers; among whom I may mention such as memory recalls—Rigby, Ramsbotham, Burns, Barnes and Leishman; Dubois, Chailly, La-Chapelle and Cazeaux; Naegele, Stoltz, Siebold, Grenser and Kolb; Dewees, Meigs and Thomas.

Of all writers, however, no one is so emphatic in his indorsement of the relation of the placenta to the os internum, as first given by Portal, as Cazeaux. He even adopts a new phraseology; his expression for P. prævia being: "The insertion of the placenta upon the lower segment of the uterus." And in beginning his chapter on the treatment, he calls the attention of his readers to what he considers the true anatomy of the cervix, up to two weeks of term, repeating his well known opinions, that up to that period the neck is entirely undeveloped, and has taken no part in forming the cavity of the uterus. In the opinion of the mass of obstetrical writers, then, the site of the placenta when prævia is *above the os internum.* This I regard as a great error. The placenta, in placenta prævia centralis, according to my views, is *below the os internum.* It rests upon the lower portions of the developed infra- and supra-vaginal portions of the cervix, and is therefore situated *between* the os externum and internum, above the one and below the other. And its presence in this locality is easily accounted for. Observation teaches that while some portion of the fundus uteri is the locality intended by nature for the rest and growth of the ovum, there is no vital or mechanical necessity that it should grow there, and there only. On the contrary, at what spot the ovulum shall rest and take root seems, in no small degree, to be left to chance. Thus, it may remain in the ovary, or it may develop in any portion of the fallopian tubes, or even outside of the containers intended for it, in the general cavity of the abdomen.

It is generally believed that an ovule which meets no fecundating fluid, passes through the uterine passage out of the body. Now, I regard placenta obvia as what Cazeaux, with his ideas as to what constitutes the uterine cavity proper during pregnancy, might view as an extra-uterine fœtation. It is a case in which the ovum has passed through the cavity of the uterus, and lodged and developed in the cavity of the neck. We may suppose the ovum to have been impregnated at the usual locality, in the ovaries, and to have escaped lodgment till the cervix was reached, or we may conceive that it had proceeded as far outward as the cervical cavity before impregnation, or perhaps an ovulum which had but just taken root in the body of the uterus, may have been expelled from that cavity, and have replanted itself within the cervix.

It may be stated, as an objection to this theory, that the ovule passing toward the uterus, from the fallopian tubes, would be estopped from entering the uterine cavity proper by the decidua reflexa of Hunter. I beg leave to call the attention of any member, who may have failed to note the advances in the study of the physiology of the impregnated uterus, to the fact that it is now generally conceded that the fallopian tubes are not occluded by the decidua, and that the ovum enters the uterine cavity uncovered by this membrane, and is so far free to pass on through the os interum.

Once in the cervix, the manner in which the ovum engrafts itself on the walls of that canal may be supposed to determine the form of the subsequent placental attachment; thus, if it grow to one circumference of the tube there will result a placenta lateralis, more or less pronounced, according as the arrest of the egg takes place, at a lower or higher point in the cavity of the neck. If the ovule attach itself to the entire circumference of the cervical canal, there results a placenta centralis.

There are two striking facts in the history of placenta prævia which demand consideration in estimating the value of any theory of its causation, namely: first, a tendency to the recurrence of the accident in certain women; second, the occasional apparent *prevalence* of the condition. Instances have been noted in which placenta oblata has occurred in the same patient in two, three, or ten successive pregnances. Cases seem occasionally to occur *en masse,* so as to lead some writers to speak of the abnormal presentation of the after-birth as almost epidemic. For example, in the early practice of Prof. W. H. Byford, no cases occurred for a series of years, when he was called in one night to three cases. The two circumstances just stated, in the record of the anomaly under consideration, indicate that among its causes is some condition competent to affect a community. The condition peculiar to the woman might be, among others, the character of the membrane of the uterus, permitting the easy passage over it of the ovum, coupled with a patency of the os internum, or a degree of irritability of the body of the womb sufficient to thrust the ovule onward. Possibly, in some cases the individual susceptibility might be found in the habit of avoiding sexual intercourse for a sufficient number of days after menstruation to permit of the arrival of the egg in the cavity of the neck before impregnation. Among the agencies competent to affect numbers of women, might be considered any cause capable of exciting an unusual uterine irritability. It is well known that in times of general excitement, anxiety, and alarm, as during the siege of a city, abortions and miscarriages are so frequent as almost to be considered epidemic. *A fortiori*, a similar influence might result in the extrusion of the ovum from the contractile uterus to rest in the less unquiet neck.

If the theory of the fore-coming after-birth here set forth be correct, it might reasonably be expected that the development of the ovum, at so low a point in the womb, would give rise to certain peculiarities by which the existing condition might be recognized. Thus, Cazeaux states that a case of placenta prævia was diagnosed at five months on account of the whole pelvic circle, as felt within the vagina, being filled with substance having a soft feel, like that of the uterus at three months. In a case of placenta prævia, recently occurring in my practice, I was consulted a number of times from the fifth to the seventh month, on account of the very low position to which, I was told, the womb had fallen. At the seventh month the vulva was pressed upon and somewhat dilated. At seven and one-half months, however, when an opportunity to see the patient was had, I was told that the uterus had lately risen, and was then giving no trouble. In this case it is probable that an early examination would have revealed such a condition of the parts as might have led to a correct diagnosis.

Madame LaChapelle is quoted by Cazeaux as recognizing, in several instances, an intra-cervical variety of placenta prævia. Leishman quotes Tyler Smith as saying that the impregnation of the ovule may take place as low in the uterus as the cervix. Dr. Smith, while he uses the language quoted by Leishman, does not seem to speak of the ovule developing in the cervix as Leishman supposes. The latter, however, seems to have conceived the idea of such development from Dr. Smith's language, and he accepts the theory of the growth of the ovum within the cervix as a satisfactory explanation of the phenomenon of placenta prævia. It must be borne in mind, however, that Dr. Leishman, even while he receives the intra-cervical growth of the ovum as explanatory of the existence of placenta prævia, never seems to conceive of the placenta when prævia in any other position than out of the cavity, to-wit: above the os internum.

To a full and consistent adoption of the intra-cervical theory of placenta prævia here given, it is essential that the premises of that theory shall be accepted, and that the placenta shall not at one moment be referred to as within the cervix, and in the next sentence be located above the os internum.

In my opinion, the subject will never be properly understood till the anatomy of the neck of the uterus, before, during and after labor, as commonly received, with all its glaring inconsistencies, be entirely abandoned, and juster conceptions of the true part which the cervix uteri, the os internum, and the os externum play in parturition, in labor, and in involution, be better understood.—*Chicago Med. Jour. & Ex.*

